# Mining the Hidden Pharmacopeia: Fungal Endophytes, Natural Products, and the Rise of AI-Driven Drug Discovery

**DOI:** 10.3390/ijms27031365

**Published:** 2026-01-29

**Authors:** Ruqaia Al Shami, Walaa K. Mousa

**Affiliations:** 1 College of Pharmacy, Al Ain University, Abu Dhabi 64141, United Arab Emirates; ruqaia.alshami@aau.ac.ae; 2 Department of Pharmacognosy, Faculty of Pharmacy, Mansoura University, Mansoura 35516, Egypt

**Keywords:** natural products, AI-enabled drug discovery, fungal endophytes, active metabolites

## Abstract

Emerging from millions of years of evolutionary optimization, Natural products (NPs) remain unique, unparalleled sources of bioactive scaffolds. Unlike synthetic molecules engineered around single therapeutic targets, NPs often exhibit multi-target, system-level bioactivity, aligned with the principles of network pharmacology, which modulates pathways in a coordinated, non-disruptive manner. This approach reduces resistance, buffers compensatory feedback loops, and enhances therapeutic resilience. Fungal endophytes represent one of the most chemically diverse and biologically sophisticated NP reservoirs known, producing polyketides, alkaloids, terpenoids, and peptides with intricate three-dimensional architectures and emergent bioactivity patterns that remain exceptionally difficult to design de novo. Advances in artificial intelligence (AI), machine learning, deep learning, and multi-omics integration have redefined the discovery landscape, transforming previously intractable fungal metabolomes and cryptic biosynthetic gene clusters (BGCs) into tractable, predictable, and engineerable systems. AI accelerates genome mining, metabolomic annotation, BGC-metabolite linking, structure prediction, and activation of silent pathways. Generative AI and diffusion models now enable de novo design of NP-inspired scaffolds while preserving biosynthetic feasibility, opening new opportunities for direct evolution, pathway refactoring, and precision biomanufacturing. This review synthesizes the chemical and biosynthetic diversity of major NP classes from fungal endophytes and maps them onto the rapidly expanding ecosystem of AI-driven tools. We outline how AI transforms NP discovery from empirical screening into a predictive, hypothesis-driven discipline with direct industrial implications for drug discovery and synthetic biology. By coupling evolutionarily refined chemistry with modern computational intelligence, the field is poised for a new era in which natural-product leads are not only rediscovered but systematically expanded, engineered, and industrialized to address urgent biomedical and sustainability challenges.

## 1. Introduction

NPs have shaped therapeutic innovation for millennia [1], beginning with ancient remedies derived from diverse biological resources [2] and continuing into the modern era with the discovery of transformative drugs such as penicillin, streptomycin, cyclosporine, lovastatin, and paclitaxel. Between 1981 and 2019, more than half of all approved small-molecule drugs were NPs or their inspired derivatives [1,3]. These molecules possess defined stereochemical and architectural features, such as rigidity, three-dimensional scaffolds, and densely functionalized motifs, that remain difficult to reproduce through de novo chemical design [4]. Their complexity reflects evolutionary optimization under ecological pressures, giving rise to sophisticated modes of action that often modulate multiple biological pathways rather than targeting a single one [5]. This inherent multi-target activity aligns closely with network pharmacology, helping to explain why natural-product-derived compounds exhibit higher clinical success rates than fully synthetic small molecules [6,7].

Fungi, yeasts, and bacteria collectively produce a wide array of bioactive metabolites with antibacterial, anticancer, antifungal, antiviral, immunosuppressive, antiparasitic, and agrochemical activities [5]. Their extraordinary ecological adaptability from polar regions and deep-sea sediments to deserts, radioactive environments, and geothermal springs drives the evolution of novel metabolic strategies and chemically diverse metabolites not observed in other organisms [5]. Among these microbes, fungi stand out for their remarkable ability to occupy diverse niches and generate structurally unique metabolites through elaborate BGCs [6,7]. These pathways encode alkaloids, polyketides, terpenes, flavonoids, peptides, and numerous hybrid structures with significant pharmacological potential. To date, more than 30,000 fungal metabolites have been isolated [8]. Since the discovery of penicillin in 1928, fungi have yielded thousands of bioactive metabolites and numerous clinically important agents. These include lovastatin [8]; cephalosporins and other β-lactams; immunosuppressants such as mycophenolic acid and cyclosporine A [9]; antifungal drugs such as griseofulvin [10], caspofungin [11], micafungin [12], and anidulafungin [13]; and immunomodulators such as fingolimod [14].

A particularly rich and still underexplored source of fungal metabolites is endophytic fungi, which reside within plant tissues [15]. These organisms maintain a symbiotic association with their hosts, experiencing selective pressures and biochemical cues that differ markedly from those encountered by free-living fungi. This intimate ecological interplay drives the evolution of unique biosynthetic pathways and metabolites that are often structurally and functionally distinct from canonical fungal products [16]. Endophytes, therefore, represent an attractive reservoir for the discovery of new NPs with therapeutic and agricultural applications.

Despite their historical value, NPs’ research experienced a marked decline in the early 2000s. This downturn was driven by the pharmaceutical industry’s shift toward high-throughput synthetic chemistry, coupled with frequent rediscovery of known scaffolds and limitations in analytical technologies [17]. These challenges reduced industrial investment in natural-product pipelines, even as ongoing analysis continued to highlight the superior biological performance of NP-based scaffolds. NPs evolved under ecological competitive conditions, endowing them with mechanisms such as multi-target engagement, metabolic stability, and structural features optimized for interacting with complex biological networks [18]. Their privileged scaffolds have remained central to medicinal chemistry, with more than 70% of first-in-class small-molecule drugs approved between 1999 and 2022 retaining clear NP-derived architectural motifs [19].

In recent years, however, natural-product discovery has undergone a major resurgence, catalyzed by the emergence of AI and the proliferation of large-scale genomic, metabolomic, and multi-omics datasets. Several recent reviews highlight how machine learning and deep learning are transforming natural-product research into a predictive and design-driven discipline, enabling deeper exploration of biosynthetic diversity across microbes, plants, and complex microbiomes [19]. In organisms such as fungi and bacteria, the organization of specialized metabolite pathways into BGCs provides a tractable genomic entry point for discovery. More than 2500 BGCs and their corresponding metabolites have been experimentally characterized, and computational genome mining now enables the identification of millions of additional candidate pathways with the potential to encode novel scaffolds [17].

AI integrates seamlessly with these resources, enabling a new level of predictive power in natural-product discovery. Machine-learning and deep-learning models now support precise BGC detection, classification, and prioritization; high-throughput metabolomic annotation and dereplication; structural prediction directly from BGC architecture; multi-omics correlation for pathway identification; and computational strategies for activating silent pathways [20]. Generative AI and diffusion models allow in silico design of NP-inspired scaffolds with biosynthetic feasibility, while advances in protein-structure prediction, including AlphaFold2, open the door to rational modification of biosynthetic enzymes and pathway engineering [2].

For industry, this computational transformation offers shorter discovery cycles, improved hit-to-lead progression, stronger intellectual-property positioning, and a more promising starting point for biological experimental validation [21,22,23]. For funding agencies, the convergence of AI and NPs directly aligns with priority missions such as combating antimicrobial resistance, developing next-generation oncology therapies, improving plant resilience, and advancing microbiome-based treatments.

In this review, we survey the diverse chemical classes of NPs isolated from fungal endophytes, highlighting key structures, biosynthetic origins, and biological activities. We then present the major AI tools currently available to accelerate natural-product discovery across genome mining, metabolomics, structure prediction, multi-omics integration, and generative design. Finally, we discuss the challenges, gaps, and future opportunities at the interface of fungal NPs and AI-enabled discovery, outlining a roadmap for building a predictive, scalable, and innovation-driven natural-product research ecosystem.

## 2. Endophytes: Definition, Characteristics, and Diversity

Endophytes are a polyphyletic group of microorganisms known for their ability to colonize plant tissues without causing any harmful disease manifestations in their host plants [24]. These plant-associated microorganisms form a ubiquitous symbiotic relationship with the host plant that is generally harmless, unobtrusive, and established entirely within host tissues [25]. In these associations, the host plant provides the microorganisms with essential elements for survival, including sugars and carbohydrates. Endophytes, in turn, produce a wide range of chemical compounds known as secondary metabolites that help the host plant conserve water, absorb nutrients from the soil, withstand biotic and abiotic stresses, and protect against environmental threats. Furthermore, these metabolites contribute to plant defense against pathogens, herbivores, and insects that can affect the plant life cycle [26,27]. Owing to their continuous adaptation within this dynamic relationship, which is shaped by environmental and ecological factors, endophytes are considered a diverse and largely untapped reservoir of bioactive metabolites that can be exploited as therapeutic agents against a wide range of disease targets [28].

Among the different types of endophytes, fungi are considered the predominant microorganisms in terms of culturable biomass [26]. Multiple studies have shown that endophytic fungi are found in almost all plants growing across diverse climatic zones, reflecting their significant ecological roles within host plants [29,30]. Endophytic fungi have been isolated from a wide range of plant species, including grasses, marine plants, mosses, ferns, trees, and shrubs [31]. It has been estimated that more than one million species of endophytic fungi exist; however, less than 16% of these species have been discovered, highlighting the need for further investigation [4,32]. Endophytic fungi have been recognized as a historically important source of novel compounds, providing valuable insights into natural-product discovery. Compounds derived from fungal endophytes have demonstrated significant biological activities, including anticancer, antibacterial, antifungal, anti-inflammatory, antioxidant, neuroprotective, and antidiabetic properties [4]. Taxol is one of the most well-known FDA-approved anticancer drugs and was isolated from the endophytic fungus *Taxomyces andreanae* in 1993. Its discovery stimulated substantial scholarly interest in the untapped metabolic capacity of endophytic fungi and their unique potential to address challenges such as drug resistance [33].

The diversity of endophytic fungal taxa among different plants exhibits a persistent pattern toward certain genera and is not randomly distributed. Despite hundreds of endophytic fungal genera reported in multi-host isolation studies, most endophytic fungi belong to a relatively stable group that includes *Penicillium*, *Fusarium*, *Colletotrichum*, *Bipolaris*, *Curvularia*, *Lasiodiplodia, Chaetomium*, *Aspergillus*, *Alternaria*, and *Diaporthe*. The genus *Pestalotiopsis*, along with related taxa such as *Cladosporium, Nigrospora*, *Xylaria*, and *Trichoderma*, has also been frequently documented in these studies [34,35]. The recurrent appearance of these genera, despite their presence in unrelated plants across diverse geographical ecosystems, indicates that endophytism is shaped more by ecological traits than by host specificity. This highlights that the enrichment of these genera is associated with ecological flexibility, including tolerance mechanisms toward host defense compounds, and the presence of unique BCGs capable of encoding the synthesis of diverse bioactive metabolites with promising activities [36]. Mapping the taxonomic core of these genes is essential for linking their community structure to their unique chemical output, providing valuable insights for metabolite isolation using genome mining and predictive AI-based computational tools.

## 3. Unveiling the Metabolic Capacity of Endophytic Fungi

Among the full spectrum of biological taxa, endophytic fungi produce a diverse array of chemically distinct secondary metabolites that belong to major biosynthetic families, including alkaloids, terpenes and terpenoids, polyketides, flavonoids, and non-ribosomal peptides, each synthesized through distinct biological pathways. These metabolites have demonstrated remarkable biological activities with a wide range of pharmacological effects, encompassing anticancer, antimicrobial, antioxidant, anti-inflammatory, and neuroprotective properties [15]. Compounds such as vinblastine, vincristine, griseofulvin, retapamulin, fusidic acid, echinocandinB0, and ibrexafungerp, which was FDA-approved as an antifungal in 2021, provide strong evidence of the ability of endophytic fungi to produce therapeutic compounds that can be used directly or indirectly in the treatment of diverse diseases [15].

Additionally, the biological potential of endophytic fungi extends beyond therapeutic applications, as they have been identified as a valuable source of enzymes, including proteases, amylases, catalases, and lipases, which can be employed in biotechnological and industrial applications. Furthermore, endophytic fungi have been utilized to address agricultural and pharmaceutical challenges globally [15].

The biosynthesis of secondary metabolites is governed by BGCs, which encode enzyme families such as polyketide synthases and non-ribosomal peptide synthetases. These enzymes operate through a complex series of reactions that determine the chemical structure and pharmacological properties of the resulting compounds [37]. The sophisticated logic of these enzymatic pathways explains why similar pathways often produce structurally related secondary metabolites and underscores the importance of variations within these pathways to generate unique scaffolds with novel properties [38]. The synthesis of these compounds can also be influenced by external environmental factors, epigenetic modifications, symbiotic interactions, and biotic and abiotic stresses [38].

**Figure 1:**
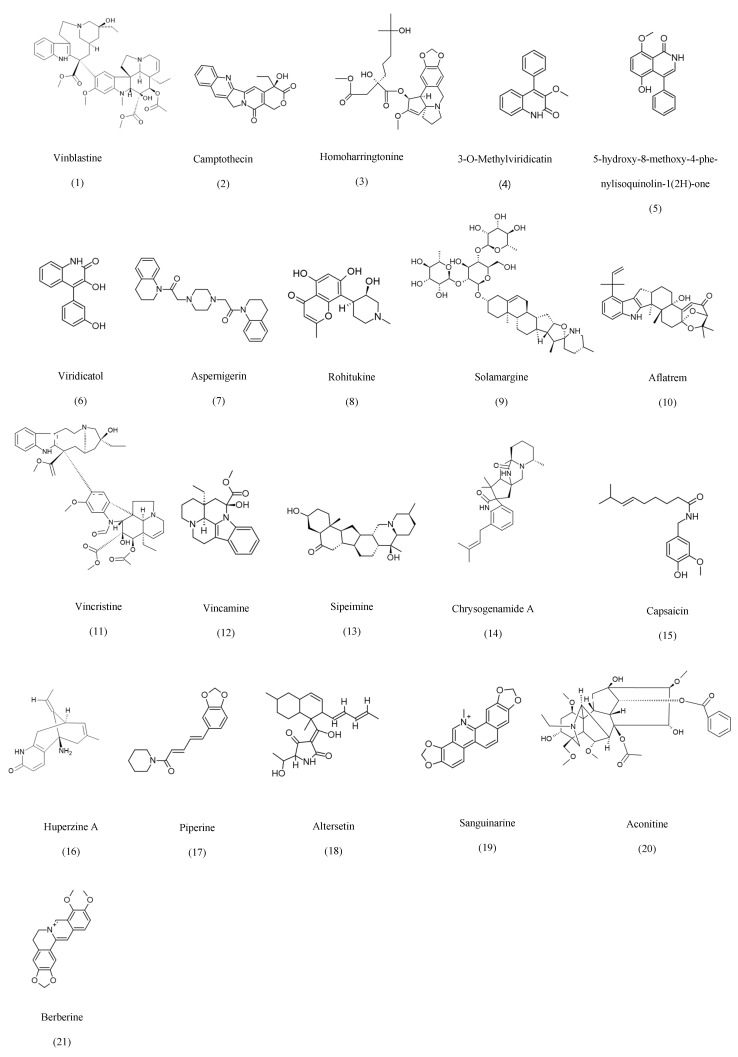
Examples of some of the alkaloids isolated from endophytic fungi.

**Figure 2:**
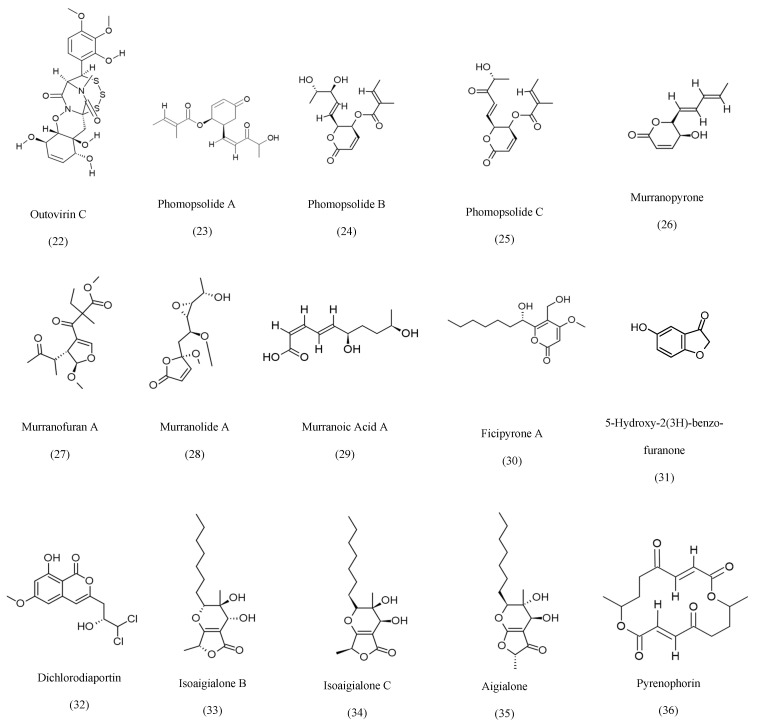

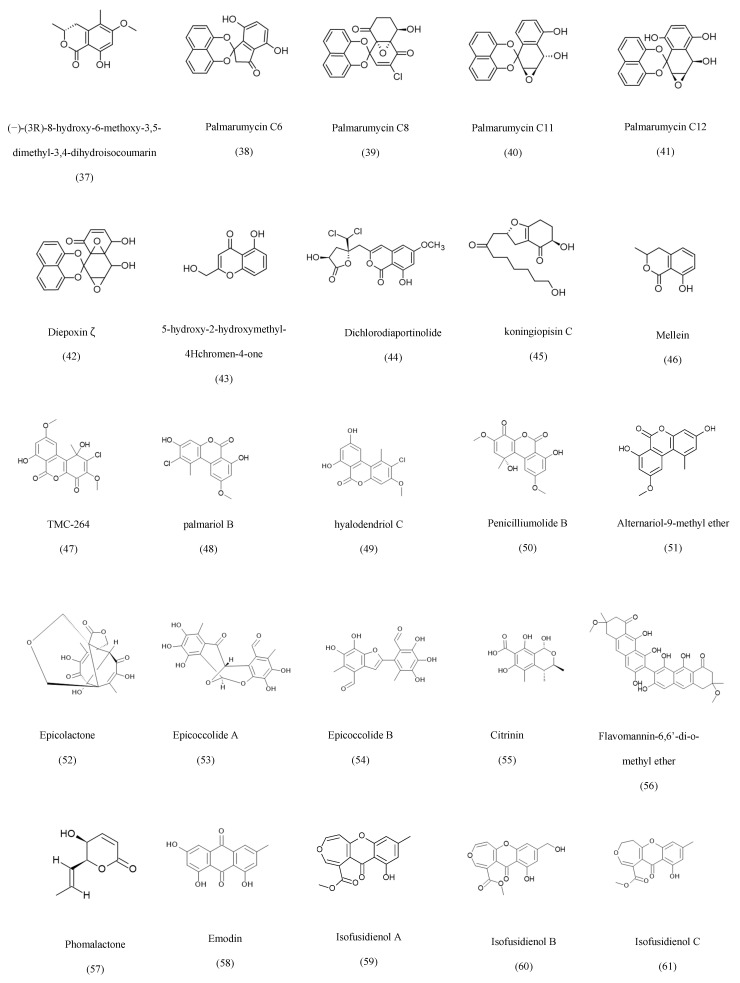

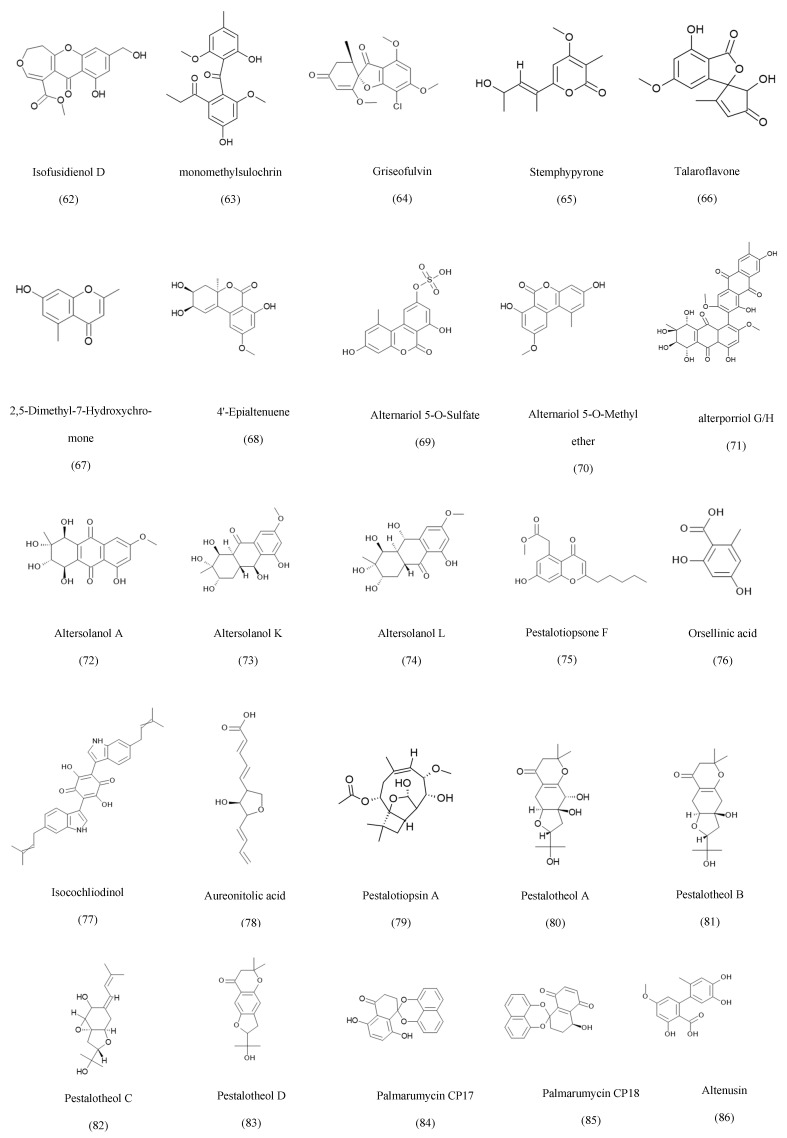

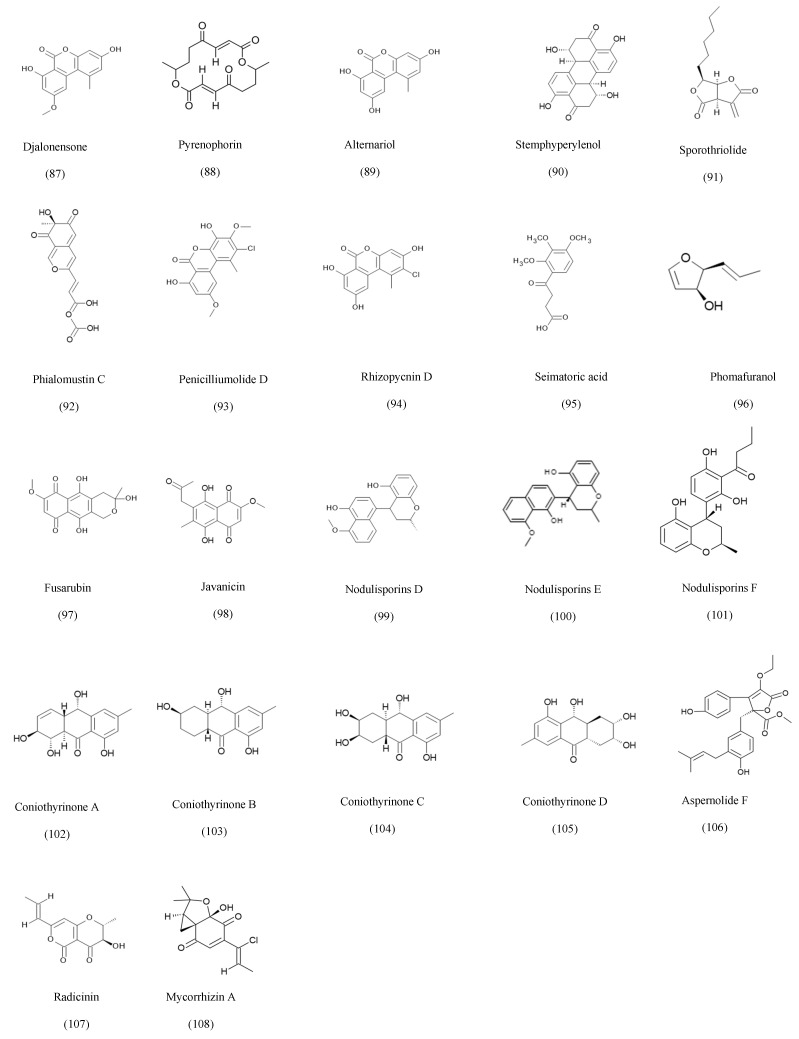
Representative examples of polyketides isolated from endophytic fungi.

**Figure 3:**
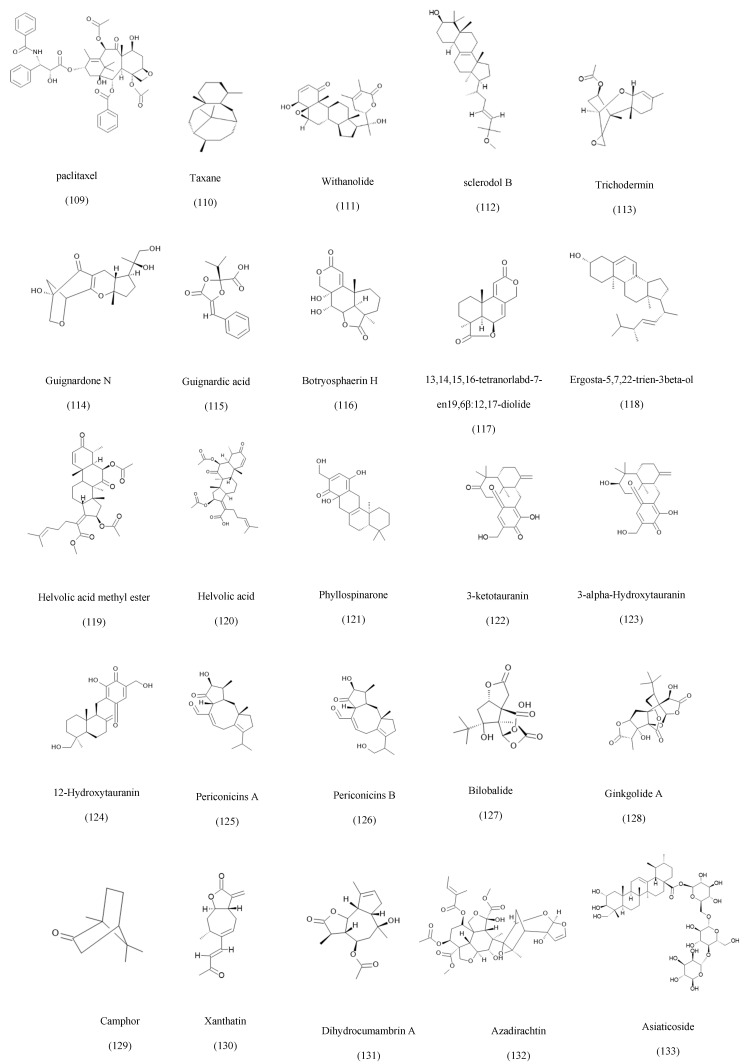

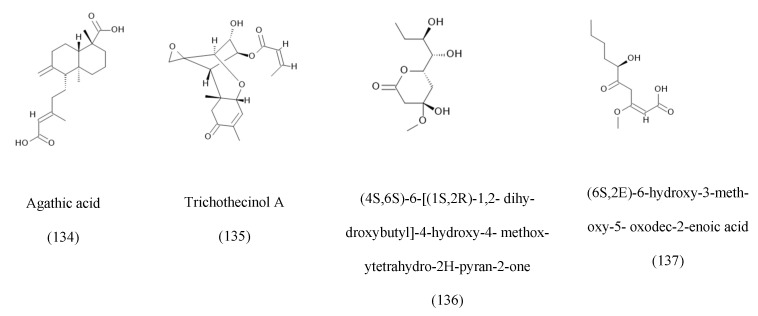
Examples of the terpenes and terpenoids isolated from endophytic fungi.

**Figure 4:**
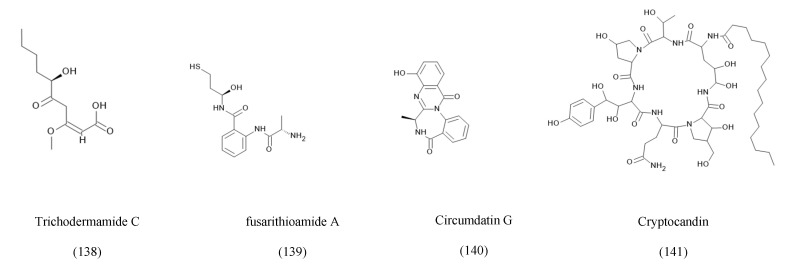
Examples of non-ribosomal peptides isolated from the co-culturing of endophytic fungi.

**Figure 5:**
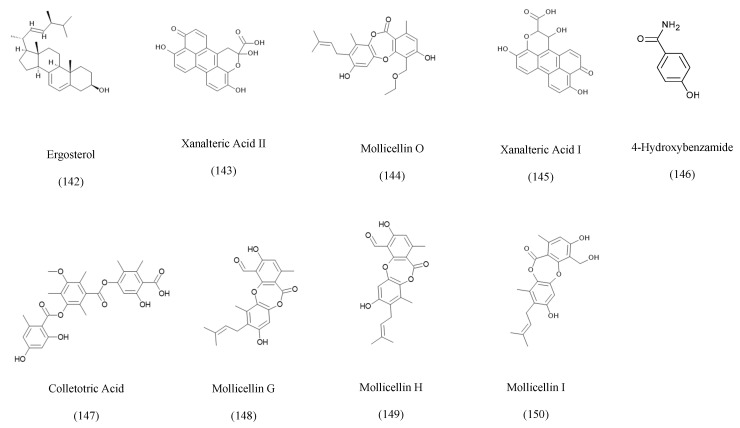
Examples of phenolics and aromatic compounds isolated from the co-culturing of endophytic fungi.

**Figure 6:**
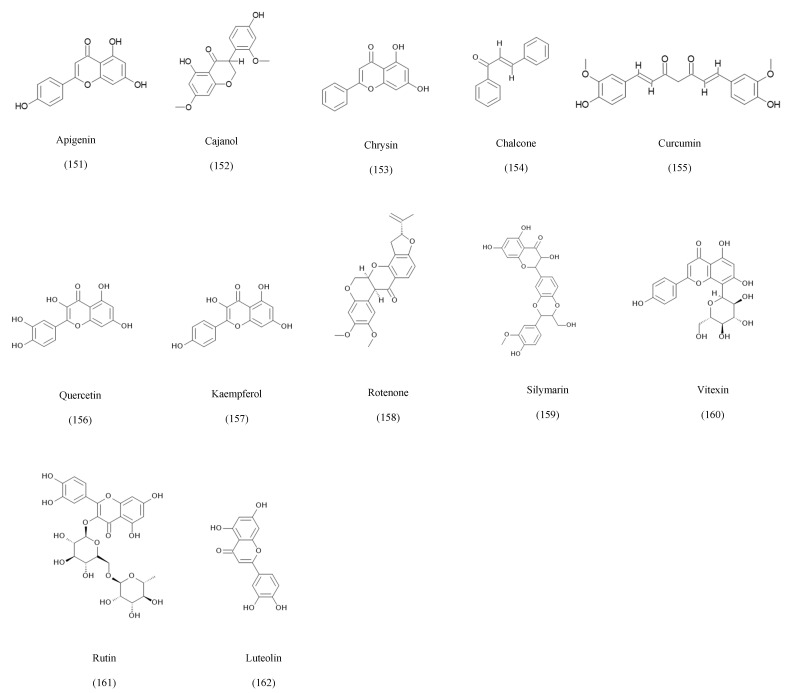
Examples of some of the flavonoids isolated from endophytic fungi.

**Figure 7:**
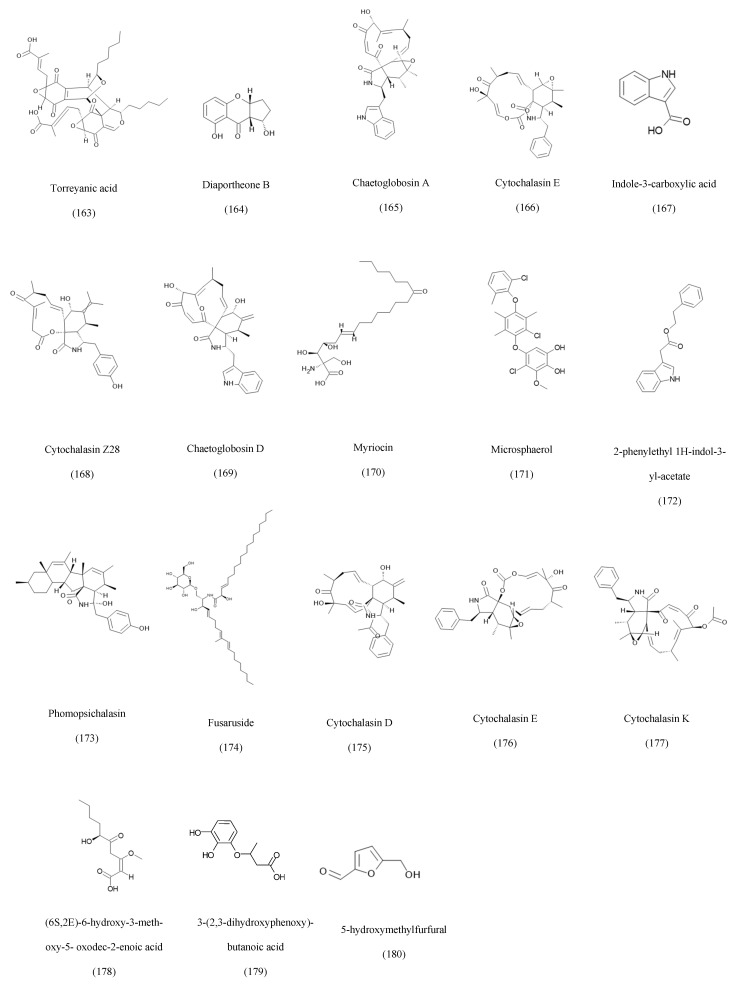
Examples of hybrid isolated compounds from endophytic fungi.

The attribution of certain fungal metabolites, such as Taxol and vinblastine, to their host plants rather than endophytic fungi remains controversial due to the potential for horizontal gene transfer, host-plant contamination, and translocated metabolites. To address these concerns, multiple studies have employed strategies such as genomic and proteomic analyses to identify specific BCGs associated with endophytic fungi, repetitive labeled isolations, and validated production evidence from axenic fungal cultures [24]. In the case of Taxol, multiple fungal species have demonstrated the ability to produce the compound across successful generations in the absence of the host plant [39]. Furthermore, several studies have suggested that the ability of some endophytes to produce the same metabolites as their host plants may result from long-term co-evolution and horizontal gene transfer [24,40]. Accordingly, the relationship between each endophyte and its host plant should be carefully examined through genomic and biochemical validation. Based on their metabolic biosynthesis, known fungal secondary metabolites can be grouped into eight categories, as compiled in Table 1, with detailed structures presented in Figure 1, Figure 2, Figure 3, Figure 4, Figure 5, Figure 6 and Figure 7.

### 3.1. Alkaloids

Alkaloids are characterized by diverse nitrogen-containing scaffolds derived from amino acids, polyketide-alkaloid hybrids, and complex heterocyclic frameworks [163]. Fungal endophytes produce numerous clinically relevant alkaloids, which are traditionally associated with their host plants, demonstrating extensive metabolic convergence within the symbiosis. For example, *Fusarium oxysporum* isolated from *Catharanthus roseus* yields the anticancer alkaloids vinblastine and vincristine [41], while *E.infrequens* produces the topoisomerase inhibitor camptothecin [164]. Several endophytes synthesize potent cytotoxic or neuroactive agents, including homoharringtonine from *Alternaria tenuissima* [42], aspernigerin from *Aspergillus niger* [45], and the tremorgenic mycotoxin aflatrem [48]. Additional nitrogenous scaffolds with antimicrobial or anti-inflammatory actions include 3-O-methylviridicatin from *Penicillium* sp. [43], viridicatol from *Phoma* sp. [44], and solamargine from *Aspergillus flavus* [47]. Endophytes also produce plant-associated alkaloids with expanded bioactivity profiles, such as rohitukine from *Fusarium oxysporum* [46], vincamine [49], and huperzine A from Colletotrichum and Trichoderma species [53]. Broad-spectrum antimicrobial and anticancer compounds such as piperine (*Colletotrichum gloeosporioides*, [54], sanguinarine (*Fusarium proliferatum* [58,59], aconitine (*Cladosporium cladosporioides*, [60], and berberine (*Alternaria* sp., [50]). Collectively, these examples highlight the enormous chemical diversity of fungal alkaloids and their ability to mirror the therapeutic potential of plant-derived metabolites. As an example, for structure activity association, Vinblastine’s core of activity is located within its dimeric structure, which is composed of catharanthine and vindoline units. The C-16 methyl ester and the nitrogen atom within the indole ring are essential for the binding affinity toward tubulin, and any further modification on them will lead to the entire loss of antinoplastic activity. Moreover, the hydroxyl group at C-3 and the ethyl group at C-4 are essential for stabilizing the protein-drug complex [165]. Campothecin inhibits Topoisomerase I through a pentacyclic planar ring system. The presence of the E-ring lactone is important for proper interaction with DNA. It is worth noting that if this ring is opened, the activity will be completely lost. The C-20 hydroxyl group (S) is essential for activity, as the (R) enantiomer is not biologically active [166]. Critical analysis of the structures of these compounds is essential to define chemical features associated with biological activity. Figure 1 shows examples of alkaloids that have been isolated from endophytic fungi.

### 3.2. Polyketides

Polyketides constitute one of the most structurally diverse classes of metabolites produced by endophytic fungi, arising from iterative polyketide synthases that generate aromatic, reduced, or highly oxygenated scaffolds [167]. Endophytes frequently biosynthesize unique polyketides not commonly encountered in free-living fungi, reflecting adaptation to their host environment and selective ecological pressures. Among the notable representatives is outovirin C, produced by *Penicillium raciborskii* from *Rhododendron tomentosum*, an antifungal agent with a polyoxygenated aromatic scaffold [61]. Members of the phomopsolide family (A–C), isolated from *Diaporthe maritima* of *Picea* sp., display characteristic butenolide motifs and exhibit diverse bioactivities [62]. Similarly, *Curvularia* sp. inhabiting *Murraya koenigii* produces the murranopyrone, murranofuran, murranolide, and murranoic acid series, highly functionalized polyketides reflecting complex oxidative tailoring reactions [63]. Several endophytes produce benzofuranone and isocoumarin structures, such as *Fusarium fujikuroi* [64,65], *Lophodermium* sp. generating pyrenophorin [68], and *Biscogniauxia mediterranea*, yielding methoxy isocoumarin derivatives [69]. The palmarumycin family biosynthesized by *Berkleasmium* sp. and *Edenia* sp. is distinguished by spirobisnaphthalene cores and demonstrates potent antiparasitic and cytotoxic properties [70,86]. Additional noteworthy polyketides include dichlorodiaportin and dichlorodiaportinolide from *Trichoderma* sp. [66], isoaigialones from *Phaeoacremonium* sp. [67], ficipyrone A from Pestalotiopsis fici [64], and mellein from *Pezicula* sp. [72]. Figure 2 represents examples of polyketides that have been isolated from endophytic fungi.

For structural analysis, Outovirin C is a secondary metabolite derived from epipolythiodioxopiperazines (ETPs). It is known for its unique sulfur-bridged diketopiperazine core. This rigid core is essential for target selectivity. The polydisulfide bridge across the diketopiperazine ring is essential for the biological activity of the compound [61].

Phomopsolide A is a potent metabolite known for its antibacterial and anticancer activity. The primary pharmacophore for this compound is the dihydropyranone ring, which allows the compound to covalently bind to the nucleophilic sites of the targeted enzyme, acting as an electrophilic Michael acceptor. The (S) configuration of the tigloyloxy group at C-5 is necessary for optimum target selection. The presence of a hydroxyl group at the pentyl chain was essential to water solubility [62].

### 3.3. Terpenes and Terpenoids

Terpenes and terpenoids are distinguished by their isoprenoid origins and extensive structural diversity, ranging from simple monoterpenes to highly oxygenated diterpenoids, sesquiterpenoids, and triterpenoid derivatives [168]. Among the most celebrated examples is Taxol (paclitaxel), a complex diterpenoid traditionally associated with *Taxus* species but now well documented in numerous endophytes, including *Aspergillus*, *Cladosporium*, *Xylaria*, *Trichoderma*, *Pezicula*, *Fusarium solani*, and *Paraconiothyrium brasiliense* [100]. These taxol-producing endophytes highlight the metabolic convergence between fungal and plant biosynthetic pathways. Related taxane-type diterpenoids have also been isolated from *Alternaria* sp. associated with *Taxus baccata* and from multiple other genera inhabiting *Wollemia nobilis* [99,165]. Endophytic fungi further yield a wide spectrum of sesquiterpenoids and meroterpenoids with diverse bioactivities. For example, *Talaromyces pinophilus* from *Withania somnifera* produces withanolide derivatives [104], while *Scleroderma* sp. generates sclerodol A and B, sesquiterpenoids with potent antifungal activity [105]. Additional examples include trichodermin from *Trichoderma brevicompactum* [106], guignardone N from *Guignardia* sp. [107], and botryosphaerin H from *Botryosphaeria* sp., associated with *Huperzia serrata* [108]. Additional terpenoid metabolites isolated from endophytes include zonarene-type compounds from *Pestalotiopsis foedan* [111], phyllospinarone from *Phyllosticta spinarum* [113], periconicins A and B [169], and neuroactive bilobalide and ginkgolide derivatives from *Pestalotiopsis* and *Fusarium oxysporum* associated with *Ginkgo biloba* [114,115]. Endophytes also produce simple monoterpenes such as camphor (*Nodulisporium* sp.) with antimicrobial activity [116], as well as diverse bioactive terpenoid glycosides, steroidal precursors, and trichothecene analogs, including dihydrocumambrin A [119], azadirachtin [120], asiaticoside [121], and agathic acid derivatives [122,123,124]. Some of the discovered terpenes and terpenoids isolated from endophytic fungi are shown in Figure 3. 

The structure-activity relationship of Taxol indicates that the C-13 phenylisoserine side chain is the most critical part of the structure due to its importance for microtubule stabilization. The oxetane ring at C-4 & C-5 is essential for maintaining the active conformation of the molecule [100].

Guignardone N is a fungal metabolite derived from the meroterpenoid class of compounds. A tricyclic pyrano-carbazole-like structure defines the core of activity. The presence of different oxygenation patterns defines the main active features of this molecule. Besides that, the presence of tertiary alpha hydroxy ketone is highly linked with the biological activity, as it participates in the formation of hydrogen bonds with the active site within the fungal proteins. This will lead to interference in the cell wall integrity and synthesis [107].

### 3.4. Nonribosomal Peptides (NRPs)

Endophytic fungi synthesize a diverse array of nonribosomal peptides (NRPs) and peptide-derived metabolites through multimodular nonribosomal peptide synthetases (NRPSs), which incorporate both canonical and nonproteinogenic amino acids into structurally complex frameworks [170]. These metabolites frequently display cyclic motifs, unusual heterocycles, and extensive oxidative tailoring, which contribute to potent biological activities. Representative examples include trichodermamide C from *Cryptosporiopsis quercina* inhabiting *Tripterygium wilfordii*, a peptide with notable antifungal activity [15]. *Eupenicillium* sp. isolated from *Glochidion ferdinandi* produces fusarithioamide A, a sulfur-containing peptide exhibiting strong cytotoxic effects [127]. Likewise, *Fusarium chlamydosporium* associated with *Anvillea garcinii* synthesizes circumdatin G, a diketopiperazine-type peptide displaying cytotoxic and antimicrobial properties [128]. Several NRPs also possess potent antiparasitic or antifungal activity. For example, Cryptocandin, a lipopeptidic echinocandin analog produced by *Fusarium* sp. from *Mentha longifolia*, exhibits broad-spectrum antifungal and antimalarial properties [15]. Figure 4 represents some of the key NRPs isolated from endophytic fungi.

Cryptocandin is a lipopeptide metabolite that involves a cyclic hexapeptide core structure and is attached to a lipid tail as a side chain. The core cyclic structure is essential for targeting the fungal cell wall, while the lipid tail is utilized to integrate into the lipid membrane of fungi [171].

### 3.5. Phenolic Derivatives

Phenolic and aromatic metabolites represent a structurally diverse group of endophytic fungal products typically derived from the shikimate or polyketide pathways [172]. These compounds are characterized by aromatic ring systems bearing hydroxyl, methoxy, keto, or carboxyl substituents, often accompanied by oxidative or halogenated modifications that enhance their biological activities. Endophytes such as *Chalara* sp. associated with Artemisia vulgaris produce ergosterol, a sterol-like phenolic metabolite with notable antibacterial activity [129]. Several *Alternaria* species isolated from *Sonneratia alba* biosynthesize xanalteric acids I and II, displaying antimicrobial properties [97,130]. Additional phenolic metabolites include 4-hydroxybenzamide from *Colletotrichum gloeosporioides* inhabiting *Michelia champaca*, which has potent antifungal activity, and colletonoic acid from *Penicillium chrysogenum* associated with *Cistanche deserticola*, known for its neuroprotective effects [93]. Phenolic acids and polyketide-derived aromatics such as mollicellins G–I, isolated from *Chaetomium* sp. in *Eucalyptus exserta*, exhibit antibacterial, antioxidant, and cytotoxic activities [132], while related chromone and benzopyrone derivatives from various endophytes contribute additional antifungal and cytotoxic properties. Figure 5 represents some phenolic compounds isolated from endophytic fungi.

Mollicellin G is a fungal metabolite that contains chlorine and aldehyde groups on the aromatic rings, which are critical for the antibacterial activity [132].

The free carboxylic acid group within the colletotric acid and its certain orientation on the side of the chain is the key responsible for the antimicrobial activities of this compound [93].

### 3.6. Flavonoids

Flavonoids, although primarily associated with plant biosynthesis, have increasingly been identified in endophytic fungi, suggesting metabolic convergence or horizontal enzymatic mimicry within plant–fungus symbioses [173]. These metabolites are typically characterized by their polyphenolic C6–C3–C6 frameworks and diverse substitution patterns, which confer potent antioxidant, anti-inflammatory, cytotoxic, and enzyme-modulatory activities. Among the most frequently reported endophytic flavonoids is apigenin, produced by *Colletotrichum* sp. isolated from *Ginkgo biloba*, as well as by *Chaetomium globosum* and species associated with *Cajanus cajan* and *Cephalotaxus harringtonia*, demonstrating broad antidiabetic, antioxidant, anticancer, and antibacterial effects [133,134,135,136]. Other endophytes biosynthesize structurally diverse flavonoids such as cajanol from *Hypocreales* species inhabiting *Cajanus cajan*, which exhibits significant antimicrobial and anticancer activities [137], and chrysin produced by *Alternaria alternata* from *Passiflora incarnata* as well as other *Colletotrichum* species [138]. Chalcones from *Ceriporia lacerata* associated with *Cleistocalyx operculatus* display anti-inflammatory, antibacterial, antifungal, and cytotoxic properties [139], while *Chaetomium globosum* isolated from *Curcuma wenyujin* produces curcumin, a metabolite with established antioxidant, antitumor, and anti-inflammatory activity [140]. Endophytic fungi also synthesize quercetin, kaempferol, vitexin, rutin, and luteolin, metabolites traditionally considered plant-exclusive. These include *Aspergillus nidulans*, *Annulohypoxylon* species, *Nigrospora oryzae*, and *Chaetomium* species across various hosts [135,141,142,143,144,145,146,147,148,149,150,151,152,153]. Figure 6 represents key structures of flavonoids with a variety of biological activities.

Apigenin and chrysin are flavones recognized by the C2, C3 double bond, and the C4 carbonyl group, which are essential for their antioxidant effect. Regarding activity, apigenin is considered more potent due to the presence of the 4-OH group on the B-ring that will enhance the receptor affinity [136].

Cajanol is an isoflavanone that depends on the 5-OH and 7-OMe substituting pattern on the A-ring, which is essential for the antimicrobial activity [137].

### 3.7. Steroids

Steroidal metabolites are characterized by their tetracyclic core structure and diverse oxidation, epoxidation, or side-chain modifications [174]. These metabolites frequently display potent antimicrobial, anticancer, anti-inflammatory, or immunomodulatory activities and often parallel or expand upon the chemical diversity of plant-derived steroids. Steroids mostly contribute to host defense or metabolic resilience. A well-documented example is *Aspergillus terreus* isolated from *Carthamus lanatus*, *which* synthesizes (22E,24R)-stigmasta-5,7,22-trien-3β-ol, a sterol demonstrating antimalarial, antimicrobial, and anti-leishmanial activities [98]. The rigid sterol core of the structure and 3β-OH are both essential for bioactivity and the interaction with the target. The E-Δ22 double bond and C-24 (R) alkyl group are responsible for target selectivity to interact with fungal and Gram-positive bacteria cell walls specifically, thus reducing toxicity in mammalian cells.

### 3.8. Hybrid Metabolites

Endophytic fungi produce a broad spectrum of metabolites that do not fall neatly into classical chemical categories [174]. This heterogeneous group includes azaphilones, cytochalasins, polyketide-alkaloid hybrids, lactones, sphingolipid analogs, and diverse indole derivatives. Many of these metabolites exhibit strong cytotoxic, antimicrobial, antifungal, antiparasitic, or neuroprotective activities, reflecting their ecological roles in mediating plant-fungus interactions and competition with other microbes. For example, torreyanic acid from *Diaporthe* sp. associated with mangroves, a highly oxygenated dimeric quinone exhibiting potent cytotoxicity [154]. Cytochalasin-type alkaloid-polyketide hybrids are widely produced across endophytes: chaetoglobosin A from *Alternaria* sp. [155] and *Chaetomium globosum* [158]. Cytochalasin E from *Chaetomium globosum* associated with *Panax notoginseng* [156], cytochalasin Z28 from Xylaria hypoxylon [157], and cytochalasin D from *Aspergillus clavatus* endophytic in *Taxus mairei* displaying antiangiogenic activity. These metabolites are characterized by large macrocyclic frameworks fused to isoindolone moieties, enabling strong modulation of cytoskeletal dynamics. Additional structurally unique compounds include indole-3-carboxylic acid from *Chaetomium* sp. [83], various polyketide–indole esters such as 2-phenylethyl 1H-indol-3-yl-acetate [131], and bioactive lactones like phomopsichalasin from Diaporthe sp. P133 is associated with *Pandanus amaryllifolius*, which exhibits notable antibacterial activity [161]. Fungal sphingolipid analogs such as fusaruside from *Fusarium* sp. IFB-121 [162] further demonstrates the biosynthetic versatility of endophytes. Other examples include microsphaerol from *Microsphaeropsis* sp. [160], seimatoric acid from *Colletotrichum* sp. [93], and 5-hydroxymethylfurfural from *Botryosphaeria dothidea* [88], exhibiting diverse antimicrobial, antioxidant, antifungal, cytotoxic, and phytotoxic effects. Representative structures from this category are shown in Figure 7.

The activity of torreyanic acid is attributed to its electrophilic diastereomeric epoxyquinone core structure that will undergo nucleophilic attack by cellular thiols, which will result in inducing apoptosis in cancer cell lines [175].

The diaportheone B core structure is essential for its biological activity. It is composed of a pyrone ring fused with a benzene ring. The phenolic hydroxyl group and the presence of a side chain at C-2 are the factors for the biological properties of the compound [76].

## 4. Clinical Translation Pipeline of Endophytic Fungi-Derived Natural Products

Endophytic fungi have demonstrated a remarkable capacity to produce a wide variety of bioactive metabolites with diverse chemical structures and biological activities. These metabolites arise from long-term co-evolution with their host plants, combined with ecological pressures, leading to the development of novel compounds with unconventional modes of action. This renders them promising candidates for the discovery of new clinical therapeutics [40].

### 4.1. Examples of Clinically Approved Drugs Isolated from Endophytic Fungi

#### 4.1.1. Palixital (Taxol)

Palixital is a diterpenoid-derived metabolite known for its antineoplastic activity. It promotes the assembly and stabilization of microtubules, leading to mitotic arrest in cancer cells. Palixital has been used to manage multiple malignancies, including non-small cell lung, ovarian, and breast cancers. Two decades after its discovery, Palixital was FDA-approved in 1992 for the treatment of ovarian cancer. It was first isolated from the medicinal plant *Taxus brevifolia* in 1971 and was later reported in *T. andreanae*. The discovery of Palixital led to a series of similar findings from 83 different endophytic sources isolated from various plant species [176,177,178].

#### 4.1.2. Vinblastine

Vinblastine is an alkaloid-derived compound first isolated from Catharanthus roseus. It is the second most commonly used agent in anticancer drug regimens to treat various cancers, including acute lymphoblastic leukemia and nephroblastoma. Vinblastine interferes with spindle formation and angiogenesis in cancer cells without harming healthy cells [179,180]. The isolation of vinblastine from an endophytic fungal source was first reported in 1998 from *Alternaria* sp., hosted within *C. roseus*. Moreover, numerous studies have reported its isolation from different endophytes residing within the same plant, including *F. oxysporum*, *Talaromyces radicus*, and *Eutypella* sp. [41].

#### 4.1.3. Camptothecin-Derived Analogs

Camptothecin is a potent alkaloid anticancer first isolated from Camptotheca acuminata in 1966, followed by discoveries in other plant species, including Miquelia dentata and Nothapodytes nimmoniana [164]. Camptothecin inhibits eukaryotic topoisomerase I (TopI) activity by selectively stabilizing the TopI–dsDNA complex, leading to cell death. Despite its promising activity, Camptothecin exhibits high toxicity and low water solubility, which limit its clinical application. 10-Hydroxycamptothecin (HCPT) and 9-methoxycamptothecin (MCPT) are natural analogs of Camptothecin; They retain the same activity without these limitations and have been used as precursors for the development of synthetic anticancer drugs such as topotecan and belotecan. The isolation of Camptothecin and its derivatives has been reported from more than 20 endophytic species residing within various plants, including *E. infrequens* and *Neurospora* sp. from *Nothapodytes foetida*, *F. solani* from *C. acuminata*, and *A. alternata*, *Fomitopsis* sp., and *Phomopsis* sp. from *M.dentata*. Collectively, these findings suggest that endophytic fungi could be a promising source of more efficient and safer derivatives of natural compounds [166].

## 5. Traditional Discovery Methods: Addressing Challenges and Limitations

The discovery and isolation of secondary metabolites from endophytic fungi involves a multi-step workflow rooted in classical natural-product chemistry. The process begins with isolating endophytic fungi from the inner tissues of surface-sterilized plants, followed by culturing on nutrient-rich media such as potato dextrose agar (PDA) or malt yeast agar (MYA) [181]. Once pure, isolated colonies are established, fermentation is carried out in solid or liquid media to stimulate the production of secondary metabolites. Subsequently, extraction is performed using organic solvents of varying polarity to recover crude metabolites from the cultured fungi, which are then subjected to bioactivity-guided fractionation [182]. The final step involves purification of the active fractions using various chromatographic techniques, followed by structural characterization with Nuclear Magnetic Resonance (NMR) and Mass Spectrometry (MS) [183].

Despite its considerable success over the years, this traditional workflow has faced multiple challenges, including the frequent rediscovery of known compounds rather than novel ones. In addition, many BGCs remain silent and unexpressed under standard laboratory conditions, indicating that endophytic fungi possess untapped metabolic potential that cannot be revealed in vitro. Other limitations of traditional discovery pipelines include low-yielding scaffolds, high consumption of organic solvents, and lengthy timelines. These challenges underscore the need for new approaches to accelerate natural-product discovery, given their importance and significant contributions across multiple fields [183].

## 6. From Concept to Implementation: Artificial Intelligence in Natural Product Discovery

**Figure 8:**
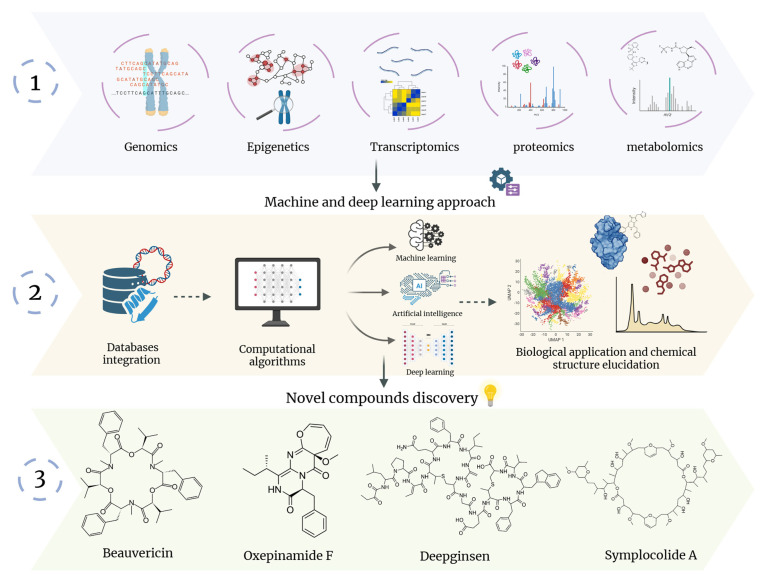
Illustration of the main NPs discovered through AI-integrated tools. (1) Represent the main types of multi-omics data that can be integrated. (2) highlight the process of data integration and processing through different approaches and their application. (3) present some of NPs that were discovered through AI algorithms.

AI is transforming NP discovery by enabling automated, high-resolution analysis of metabolomic and genomic datasets, thereby accelerating dereplication, BGC annotation, and structural prediction. AI-driven genome-mining platforms such as antiSMASH, DeepBGC, and GECCO substantially improve BGC detection, classification, and prioritization, uncovering hidden biosynthetic potential that traditional approaches often miss [184,185,186,187]. In metabolomics, tools such as GNPS molecular networking, SIRIUS/CSI:FingerID, and MS2LDA extract structural and substructural information from complex LC–MS/MS datasets, facilitating analog discovery and filtering out known compounds before time-consuming isolation steps [188]. Complementary platforms, including PRISM and NRPSPredictor2/SANDPUMA, link genomic and metabolomic information by predicting the chemical scaffolds encoded within fungal BGCs, thereby guiding targeted compound identification [185,186,187]. Deep-learning frameworks further support discovery by identifying cryptic or silent BGCs and predicting environmental or regulatory cues capable of activating these pathways in vitro, unlocking the untapped metabolic potential of endophytic fungi [24], When integrated with traditional wet-lab workflows, AI shifts NP discovery from an empirical, trial-and-error model to a predictive, design-driven pipeline, accelerating the identification of novel pharmaceutical leads [189,190]. Figure 8 illustrates how AI and machine-learning algorithms mine genomic and metabolomic data to guide the prediction and discovery of new natural-product scaffolds. A summary of major AI tools applied to NP discovery over the past decade is provided in Table 2.

### 6.1. Artificial Intelligence and Deep Learning Algorithms: Promising Tools for Natural Product Discovery

AI is recognized as a key approach for analyzing large biological datasets using machine-learning and deep-learning algorithms. These methods can predict the distribution of plant endophytes and their bioactive metabolites across diverse biological niches. The process can be optimized by incorporating initial multi-omics and metabolomic data from plants, followed by detailed integration of targeted regions through multi-omics analyses and metabolic pathway prediction tools. Currently, various machine-learning and deep-learning algorithms are being employed to enhance drug discovery from natural resources [184].

DeepBGC software (https://github.com/Merck/deepbgc, accessed on 19 December 2025) is designed to analyze large datasets from microbial communities by applying a skip-gram neural network model, similar to word2vec, to generate embeddings that are paired with a bidirectional long short-term memory (BiLSTM) network, trained on extensive datasets derived from microbial communities. Computational biology analyses and genome-mining tools emphasize the integration of various metabolomic approaches to effectively study the plant microbiome models and their associated biochemical changes, providing deeper insights into metabolic regulatory pathways and their interactions. Furthermore, by integrating deep-learning algorithms, genomic metabolic models, and cheminformatics tools, it is possible to perform detailed bio-prospective analyses of endophytes and their metabolites, efficiently predicting the chemical scaffolds of these compounds. Collectively, these deep-learning approaches and novel computational pipelines represent a significant milestone in natural-product discovery from endophytes [184].

Recent advancements in AI algorithms have unlocked the potential of generative models to predict ADMET properties for lead optimization. Generative models, such as generative adversarial networks (GANs) and variational autoencoders (VAEs), can propose structurally novel analogs by incorporating predefined optimization criteria, enabling exploration of the chemical space surrounding a scaffold of interest. These generative architectures also support de novo design with an inherent bias toward desirable structural features, which can be combined with predictive models for absorption, distribution, metabolism, excretion, and toxicity (ADMET) to eliminate unfavorable candidates early in the discovery process. Graph neural network models, when paired with ADMET predictors, have demonstrated utility in rapid initial risk assessments and prioritization of compounds based on traditional QSAR approaches, facilitating efficient screening and evaluation of in silico libraries. By integrating these predictive frameworks with ADMET models, researchers can selectively prioritize compounds with favorable pharmacokinetic and toxicity profiles, thereby reducing the number of candidates requiring experimental validation. This approach minimizes empirical lead optimization efforts and enhances the efficiency and quality of early-stage drug discovery workflows [218,219]. Furthermore, the use of a transformer-based model may improve the early stages of drug discovery through predicting different drug properties at once, including pharmacokinetics, toxicity, and drug effectiveness, to ensure a simpler discovery process. This model has been successfully applied to HIV Integrase-1 to identify promising drug candidates and exclude unfavorable ones, highlighting it as a flexible solution support AI-driven drug discovery [220].

### 6.2. Genome Mining of Endophytic Fungi: A Case Study for AI-Assisted Fungal Metabolite Discovery

Genome mining has been proposed as a core strategy for evaluating the biosynthetic potential of endophytic fungi, particularly given the significant disparity between genomic predictions and experimentally validated metabolite production. A notable example is the genome-based analysis of the endophytic fungus *Dactylonectria alcacerensis* CT-6. In this study, they used antiSMASH (version 6.1.1) for whole-genome sequencing and annotation analysis, which revealed a high abundance of BGCs, including nonribosomal peptide synthetases, terpene synthases, polyketide synthases, and hybrid pathways. The number of putative BGCs predicted through bioinformatic analysis far exceeded the number of compounds successfully isolated ex situ, as most BGCs remain silent or are lowly expressed under standard laboratory conditions [221].

Furthermore, a genome mining tools were utilized in the case of *Ascomycete* sp., an endophytic fungus isolated from *Taxus yunnanensis* known as Chinese yew, using antiSMASH software (fungiSMASH 5.1.0), which revealed 35 putative BGCs, leading to the identification of lijiquinone 1. The isolated compound showed potential anticancer and antifungal activity [222].

From a methodological perspective, these cases highlight genome mining primarily as a predictive tool rather than a definitive drug discovery strategy. Current genome mining tools rely on rule-based detection frameworks derived from conserved enzymatic domain architectures. Neural network algorithms trained to detect BGCs can identify weakly expressed clusters and classify BGCs based on learned patterns rather than fixed rules. In the case of *D. alcacerensis* CT-6, AI tools could reduce false-negative predictions, refine BGC detection, and prioritize novel predicted clusters for subsequent experimental validation. These studies demonstrate the potential of genome mining in endophytic fungi, extensively predicting silent BGCs that were previously difficult to detect. In summary, AI tools and deep-learning algorithms should be employed as complementary approaches to reduce bias and enable prioritization of large genomic datasets, rather than as replacements for experimental validation.

## 7. Limitations and Future Perspective

Despite this rapid progress, significant gaps remain. Fungal endophytes contain extensive repertoires of transcriptionally silent or condition-dependent BGCs, and current AI pipelines, though increasingly sophisticated, still struggle to distinguish which clusters are actively expressed, functionally assembled, or chemically productive under specific ecological or laboratory conditions. Much of the available training data is derived from bacterial systems, resulting in models that do not yet generalize well to fungal biosynthetic logic, particularly iterative type I polyketide synthases (PKSs) and fungal-specific hybrid PKS–NRPS assembly lines. This taxonomic bias limits accurate prediction of domain functions, tailoring steps, and final chemical products in fungal endophytes. Furthermore, high risks of overfitting, false-positive predictions, data scarcity, and limited interpretability remain major constraints for these machine-learning algorithms [223].

Current AI tools attempt to overcome these limitations through filtration of metabolic noise, curated datasets, cross-validation across heterogeneous datasets, and re-annotation using updated BGC tools; However, their analytical capability is still insufficient to reliably detect misassigned clusters, errors associated with fragmentation patterns, and inconsistencies in metabolite expression.

Metabolomics-based AI approaches also face significant challenges. Spectral libraries remain incomplete and are disproportionately enriched for well-studied bacterial or plant compounds, leaving significant gaps in coverage for fungal metabolites. Consequently, structural assignments for truly novel scaffolds often remain low-confidence, even when advanced tools such as molecular networking, fragmentation-tree prediction, or deep-learning–based spectral annotation are applied. Multi-omics integration frameworks are emerging [224]. However, comprehensive pipelines that unify BGC expression, metabolite production, regulatory signaling, epigenetic influences, and ecological context are still lacking. The absence of large-scale paired genome and metabolome datasets further constrains the development of robust AI models capable of reliably predicting chemical output directly from genomic input. Addressing these limitations will require systematic profiling of fungal endophytes across diverse hosts, environmental conditions, and growth regimes to generate the high-quality training data essential for next-generation model development.

It is worth noting that AI-guided algorithms primarily focus on achieving chemical plausibility rather than biological feasibility, which must be further validated using established biochemical data and predictive enzymatic models. AI tools rely on previously curated databases of known metabolic interaction patterns (e.g., KEGG pathways) and established enzymatic reaction rules to design retrosynthetic pathways based on structured chemistry, rather than relying on randomness. Accordingly, AI approaches integrate predicted enzymatic functionality with structural model preferences to generate selective candidates that are compatible with realistic biochemical mechanisms [225].

Looking ahead, advances in AI-based natural-product discovery are poised to reshape the landscape of fungal NP research. Foundation models trained on massive genomic, chemical, and multi-omics datasets are expected to improve BGC boundary prediction, enzyme-function annotation, substrate specificity inference, and metabolite reconstruction with far greater accuracy than current task-specific models. Generative AI architectures, including generative adversarial networks (GANs), variational autoencoders (VAEs), and diffusion models [186], will increasingly shift discovery from passive exploration to proactive design, proposing novel NP-like scaffolds with both biosynthetic feasibility and desired bioactivity profiles.

AI-guided activation strategies are also expected to advance further. Bayesian optimization [226], reinforcement learning, and regulatory network modeling can prioritize cultural conditions or genetic perturbations most likely to activate silent fungal pathways, enabling targeted elicitation rather than random stimulation. At the level of enzyme engineering, breakthroughs in protein-structure prediction, particularly AlphaFold2 [190], will support the rational redesign of PKS and NRPS megasynthases, facilitating the generation of derivative molecules or entirely new chemical architectures.

Finally, global-scale computational analyses of microbial biosynthetic potential [192] highlight the vast reservoir of unexplored chemistry encoded within fungal and bacterial genomes. When combined with AI-driven genome mining, metabolomics, and generative modeling, these resources point to a future in which the chemical diversity of fungal endophytes can be systematically accessed, predicted, and engineered. Integrating these tools into unified, data-rich discovery pipelines will be essential for realizing the full potential of endophytic fungi as a source of next-generation therapeutics and biotechnological platforms.

## 8. Conclusions

Endophytic fungi represent a remarkably rich and still underexplored reservoir of bioactive secondary metabolites with significant pharmaceutical potential. Although traditional workflows have yielded thousands of clinically relevant scaffolds, these approaches are inherently time-consuming, resource-intensive, and prone to rediscovery. AI approaches offer a transformative solution to these constraints. The evolution of AI-based frameworks capable of predicting, annotating, and even designing complex molecular scaffolds promises to reshape the landscape of fungal NP discovery.

In this review, we highlighted the growing role of AI-integrated tools in the discovery of NPs from microbial sources, highlighting endophytic fungi as a particularly promising group. By adapting AI algorithms within the endophytic fungi discovery pipeline, the integration of computational foresight with experimental validation represents a foundational step toward unlocking the full biosynthetic potential of fungal endophytes and revitalizing NPs as a central pillar of drug discovery [227].

However, current AI-derived tools are limited by incomplete endophytic fungal genomes, metabolic noise, and the low availability of experimentally validated BGC metabolite linkages. These limitations can lead to a significantly high false-positive rate due to an overemphasis on chemical plausibility rather than enzymatic pathway feasibility. Accordingly, progress in the field will depend on improving genome data quality and reducing biases associated with multi-omic integration. Therefore, AI tools should be utilized as a supportive decision-making layer rather than a standalone solution for NP discovery.

## Figures and Tables

**Table 1:** Secondary metabolites produced by endophytic fungi and their associated biological activity.

Alkaloids and Nitrogen-Containing Derived Metabolites				
**Compounds**	**Endophytic Fungus**	**Host Plants**	**Reported Biological Activity**	**References**
Vinblastine	*Fusarium oxysporum*	*Catharanthus roseus*	Anticancer activity	[41]
Camptothecin	*Entrophospora infrequens*	*Camptotheca acuminata*		[15]
Homoharringtonine	*Alternaria tenuissima*	*Cephalotaxus* sp.		[42]
3-O-methylviridicatin	*Penicillium* sp. R22	*Nerium indicum*	Antifungal activity	[43]
5-hydroxy-8-methoxy-4-phenylisoquinolin-1(2H)-one				
Viridicatol	*Phoma* sp. WF4	*Eleusine coracana*		[44]
Aspernigerin	*Aspergillus niger*	*Cynodon dactylon*	Cytotoxic activity	[45]
Rohitukine	*Fusarium oxysporum,*	*Dysoxylum binectariferum*	Anticancer and cytotoxic activities	[46]
	*Fusarium solani*			
Solamargine	*Aspergillus flavus*	*Solanum nigrum*		[47]
Aflatrem		*Many plants*	Neurotoxic activity	[48]
Vincristine	*Fusarium oxysporum*	*Catharanthus roseus*	Antitumor activity	[41]
Vincamine	*-*	*Vinca minor*	Antihypertensive activity.	[49]
Sipeimine	*Cephalosporium corda*	*Fritillaria ussuriensis*	Anti-ulcer activity	[50]
Chrysogenamide A	*Penicillium chrysogenum*	*Cistanche deserticola*	Neuroprotective activity	[51,52]
Capsaicin	*Alternaria alternata*	*Capsicum annuum*	Anti-inflammatory activity	[52]
Huperzine A	*Colletotrichum* sp. *Trichoderma* sp.	*Huperzia serrata*	Acetylcholinesterase inhibitor activity.	[53]
Piperine	*Colletotrichum gloeosporioides*	*Piper nigrum*	Anti-cancer, Anti-microbial, Anti-inflammatory, antidepressant, and hepatoprotective activities.	[54]
	*Periconia* sp.			[55]
	*Mycosphaerella* sp.			[56]
altersetin	*Alternaria* sp.	*-*	Anti-Gram-positive activity	[57]
Sanguinarine	*Fusarium proliferatum*	*Macleaya cordata*	Antimicrobial, anticancer, antioxidant, anti-inflammatory, neuroprotective, and anthelmintic activity.	[58] [59]
Aconitine	*Cladosporium cladosporioides*	*Aconitum leucostomum*	Anti-cancer, Anti-neurologic, and anti-inflammatory activity	[60]
Berberine	*Alternaria* sp.	*Phellodendron amurense*	Anti-bacterial, antihypertensive, anti-diabetic, antiproliferative, anti-hyperlipidaemic, and vasodilator activity	[50]
Polyketide-derived metabolites				
Outovirin C	*Penicillium raciborskii*	*Rhododendron tomentosum*	Antifungal activity	[61]
Phomopsolide A	*Diaporthe maritima*	*Picea* sp.		[62]
Phomopsolide B				
Phomopsolide C				
Murranopyrone	*Curvularia* sp. strain M12	*Murraya koenigii*		[63]
Murranofuran A				
Murranolide A				
Murranoic acid A				
Ficipyrone A	*Pestalotiopsis fici*	*Camellia sinensis*		[64]
5-hydroxy 2(3H)-benzofuranone	*Fusarium fujikuroi* (WF5)	*Eleusine coracana*		[65]
Dichlorodiaportin	*Trichoderma* sp. 09	*Myoporum bontioides*		[66]
Isoaigialone B	*Phaeoacremonium* sp.	*Senna spectabilis*		[67]
Isoaigialone C				
Aigialone				
Pyrenophorin	*Lophodermium nitens* DAOM 250027	*Pinus strobus*		[68]
(−)-(3R)-8-hydroxy-6-methoxy-3,5- dimethyl-3,4-dihydroisocoumarin	*Biscogniauxia mediterranea*	*Echinacea purpurea*		[69]
Palmarumycin C6	*Berkleasmium* sp.	*Dioscorea zingiberensis*		[70]
Palmarumycin C8				
Palmarumycin C11				
Palmarumycin C12				
Diepoxin ζ				
Cladospirone B*				
5-hydroxy-2-hydroxymethyl-4Hchromen-4-one	*Nodulisporium* sp.	*Erica arborea*		[15]
Dichlorodiaportinolide	*Trichoderma* sp. 09	*Myoporum bontioides*		[66]
Koningiopisin C	*Trichoderma koningiopsis*	*Panax notoginseng*		[71]
Mellein	*Pezicula* sp.	*Forsythia viridissima*		[72]
TMC-264	*Hyalodendriella* sp.	*Populus deltoides Marsh*	Antimicrobial activity	[73]
Palmariol B				
Hyalodendriol C				
Penicilliumolide B				
Alternariol 9-methyl ether				
Epicolactone	*Epicoccum* sp.	*Theobroma cacao*		[74]
Epicoccolide A				
Epicoccolide B				
Citrinin	*Penicillium* *citrinum*	*-*		[75]
Flavomannin-6,6’ -di-O-methyl ether	*Diaporthe melonis*	*Annona squamosa*		[76]
Phomalacton	*Phoma* sp.	*Fucus serratus*		[77]
Emodin	*Aspergillus glaucus*	*Ulva lactuca* (Seaweed)		[15]
Isofusidienol A	*Chalara* sp.	*Artemisia vulgaris*	Antibacterial activity	[15]
Isofusidienol B				
Isofusidienol C				
Isofusidienol D				
Monomethylsulochrin	*Aspergillus* sp. strain CY725	*-*		[78]
Griseofulvin	*Xylaria* sp. strain F0010	*Abies holophylla Garcinia hombroniana*	Antioxidant activity	[79,80]
Stemphypyrone	*Stemphylium globuliferum*	*Mentha pulegium*	Anticancer activity	[81]
Talaroflavone	*Alternaria* sp.	*Polygonum senegalense*		[15]
3′-hydroxyalternariol 5-O-methyl ether *				
Alternaric acid *				
Alterlactone *				
Altenuene *				
Altertoxin I *				
2,5-dimethyl-7-hydroxychromone				
4′-epialtenuene				
Alternariol 5-O-sulfate				
Alternariol 5-O-methyl ether				
Alterporriol G	*Stemphylium globuliferum*	*Mentha pulegium*		[81]
Altersolanol A	*Ampelomyces* sp.	*Urospermum picroides*		[82]
Altersolanol K	*Stemphylium globuliferum*	*Mentha pulegium*		[81]
Altersolanol L				
Pestalotiopsone F	*Pestalotiopsis* sp.	*Rhizophora mucronata*		[15]
Orsellinic acid	*Chaetomium* sp.	*Otanthus maritimus*		[83]
Isocochliodinol				
Aureonitolic acid				
Pestalotiopsin A	*Pestalotiopsis fici*	*Taxus wallichiana*	Anti-HIV activity	[84]
Pestalotheol A	*Pestalotiopsis theae*	*-*		[85]
Pestalotheol B				
Pestalotheol C				
Pestalotheol D				
Palmarumycin CP17	*Edenia* sp.	*Petrea volubilis*	Antiparasitic activity	[86]
Palmarumycin CP18				
2′,3′-dihydrosorbicillin(9Z,12Z)-2,3-dihydroxypropyloctadeca9,12-dienoate *	*penicillium chrysogenum*	*Cistanche deserticola*	Neuroprotective activity	[51]
Altenusin	*Alternaria* sp.	*Trixis vauthieri*	Anti-leishmaniasis activity	[87]
Djalonensone	*Botryosphaeria* *dothidea* KJ-1	*Melia azedarach*	Antioxidant, Antibacterial, cytotoxic, and Antifungal activities.	[88]
Pycnophorin				
Alternariol				
Stemphyperylenol				
Sporothriolide	*Nodulisporium* sp. A21	*Ginkgo biloba*	Antifungal, and Anti-phytopathogenic activities	[89]
Phialomustin C	*Phialophora mustea*	*Crocus sativus*	Cytotoxic, and antimicrobial activities	[90]
Penicilliumolide D				
6-methyl-1,2,3-trihydroxy-7,8-cyclohepta-9,12-diene-11-one-5,6,7,8- tetralene-7-acetamide (KL-4) *	*Aspergillus* sp.	*Gloriosa superba*		[91]
Rhizopycnin D	*Rhizopycnis vagum* Nitaf 22	*Nicotiana tabacum*		[92]
Seimatoric acid	*Colletotrichum* sp.	*Gomera*	Antibacterial and Antifungal activities.	[93]
Phomafuranol	*Phoma* sp.	*Fucus serratus*		[77]
Fusarubin	*Fusarium solani*	*Glycyrrhiza glabra*	Anti-microbial and anti-tubercular Activities	[94]
Javanicin	*Fusarium* sp.	*Piper sp. (Piperaceae*	Phytotoxic and antifungal activities	[95]
Nodulisporins D	*Nodulisporium* sp.	*Erica arborea*	Antifungal and Antialgal Activities	[96]
Nodulisporins E				
Nodulisporins F				
Coniothyrinones A	*Coniothyrium* sp.	*Salsola oppostifolia*	Antifungal, Antibacterial, and Algicidal activities	[97]
Coniothyrinones B				
Coniothyrinones C				
Coniothyrinones D				
Aspernolides F	*Aspergillus terreus*	*Carthamus lanatus*	Anti-microbial, Anti-malarial, Anti-leishmanial, and cytotoxic activities	[98]
Radicinin	*Physalospora* sp.	*-*	Antifungal, Antibacterial, and Herbicidal activities	[99]
Mycorrhizin A	*Plectophomella* sp.			
Terpenes and Terpenoids—derived metabolites				
Taxol	*Aspergillus* sp.	*Taxus chinensis*	Anticancer activity	[100]
	*Ceratobasidium* sp.			
	*Cladosporium Tenuissimum*			
	*Xylaria* sp.			
	*Trichoderma* sp.			
	*Sordaria* sp.			
	*Phomopsis* sp.			
	*Pezicula* sp.			
	*Fusarium solani*			
	*Coniothyrium diplodiella*			
	*Epacris* sp.			
	*Metarhizium anisopliae*			
	*Paraconiothyrium brasiliense*			
Taxane	*Alternaria* sp.	*Taxus baccata*		[101,102]
	*Aspergillus* sp.			
	*Beauveria* sp.			
	*Fusarium* sp.			
	*Epicoccum* sp.			
	*Geotrichum* sp.			
	*Phoma* sp.			
	*Phomopsis* sp.			
	*Gelasinospora* sp.			
	*Cladosporium* sp.	*Wollemia nobilis*		[103]
	*Langeronii* sp.			
	*Phomopsis* sp.			
Withanolide	*Taleromyces pinophilus*	*Withania somnifera*		[104]
Sclerodol A *	*Scleroderma UFSM Sc1*	*Eucalyptus grandis*	Antifungal activity	[105]
Sclerodol B				
Trichodermin	*trichoderma brevicompactum* 0248	*Allium sativum*		[106]
Guignardone N	*Guignardia* sp.	*Euphorbia sieboldiana*		[107]
Guignardic acid				
Botryosphaerin H	*Botryosphaeria* sp. P483	*Huperzia serrata*		[108]
13,14,15,16-tetranorlabd-7-en19,6β:12,17-diolide				
Ergosta-5,7,22-trien-3beta-ol	*Chaetomium cupreum* *ZJWCF079*	*Macleaya cordata*		[109]
Helvolic acid methyl ester	*Fusarium* sp.	*Ficus carica*		[110]
Hydrohelvolic acid *				
Helvolic acid				
(3R,4R,6R,7S)-7-hydroxyl-3,7-dimethyl-oxabicyclo [3.3.1] nonan-2-one *	*Pestalotiopsis foedan*	*Bruguiera sexangula*		[111]
(3R,4R)-3-(7-methylcyclohexenyl)-propanoic acid *				[112]
Phyllospinarone	*Phyllosticta spinarum*	*Platycladus orientalis*	Cytotoxic activity	[113]
3-ketotauranin				
Tauranin -(+)-(5 S,10 S)-4′-hydroxymethylcyclozonarone *				
3 alpha-hydroxytauranin				
12-hydroxytauranin				
3b-hydroxy-5a, 8a-epidioxy-ergosta-6, 22-diene *	*Aspergillus* sp. strain CY725	Not reported	Antibacterial activity	[15]
Periconicins A	*Periconia* sp.	*Taxus cuspidata*		[15]
Periconicins B				
Bilobalide	*Pestalotiopsis uvicola*	*Ginkgo biloba*	Neuroprotective activity	[114]
Ginkgolide	*Fusarium oxysporum*	*Ginkgo biloba*	Anti-inflammatory activity	[115]
Camphor	*Nodulisporium* sp.	*Lagerstroemia loudoni*	Antimicrobial activity	[116]
β-sitosterol glucoside *	*Botryosphaeria dothidea KJ-1*	*Melia azedarach*	Antioxidant, Antibacterial, and anti-fungal activities.	[88]
Xanthatin	*Paecilomyces* sp.	*Panax ginseng*	Antitumor activity	[117,118]
Dihydrocumambrin A	*Botryodiplodia theobromae*	*Dracaena draco*	Cytotoxic and Antibacterial activities.	[119]
Azadirachtin	*Penicillium* sp.	*Azadirachta indica*	Hepatoprotective and insecticidal activities.	[120]
Asiaticoside	*Colletotrichum gloeosporioides*	*Centella asiatica*	Anti-inflammatory, Immunomodulatory, and antioxidant activities.	[121]
Agathic acid	*Botryosphaeria* sp.	*Maytenus hookeri*	Anti-inflammatory and anticancer activities	[122]
	*Bionectria* sp.	*Raphia taedigera*		[123]
	*Fusarium* sp.	*Santalum album*		[124]
Trichothecinol A	*Trichothecium* sp.	*Phyllanthus amarus*	Antifungal, anticancer, and antimetastatic activities	[125]
(4S,6S)-6-[(1S,2R)-1,2-dihydroxybutyl]-4-hydroxy-4-methoxytetrahydro-2H-pyran-2-one	*Pestalotiopsis* sp. DO14	*Dendrobium officinale*	Cytotoxic and Antifungal Activities	[126]
(6S,2E)-6-hydroxy-3-methoxy-5-oxodec-2-enoic acid				
Peptides and amino acid-derived compounds				
Trichodermamide C	*Cryptosporiopsis quercina*	*Tripterigium wilfordii*	Antifungal activity	[127]
Fusarithioamide A	*Eupenicillium* sp.	*Glochidion ferdinandi*	Cytotoxic activity	[127]
Circumdatin G	*fusarium chlamydosporium*	*Anvillea garcinii*	Cytotoxic and Antimicrobial activities.	[128]
Cryptocandin	*Fusarium* sp.	*Mentha* *longifolia*	Antifungal and antimalarial activities.	[15]
Phenolic and aromatic—derived compounds				
Ergosterol	*Chalara* sp.	*Artemisia vulgaris*	Antibacterial activity	[129]
Xanalteric acid II	*Alternaria* sp.	*Sonneratia alba*		[130]
Mollicellin O				
Xanalteric acid I	*Coniothyrium* sp.	*Salsola oppostifolia*	Antimicrobial activity	[97]
4-hydroxy-benzamide	*Colletotrichum gloeosporioides*	*Michelia champaca*	Antifungal activity	[131]
Colletonoic acid *	*Penicillium chrysogenum*	*Cistanche deserticola*	Neuroprotective activity	[93]
Colletotric acid	*Colletotrichum* sp.	*Gomera*	Antibacterial, antifungal, and antialgal activities	[93]
Mollicellin G	*Chaetomium* sp.	*Eucalyptus exserta*	Antibacterial, antioxidant, and cytotoxic activities	[132]
Mollicellin H				
Mollicellin I				
Steroid-Derived Metabolites				
(22E,24R)-stigmasta 5,7,22-trien-3-β-ol *	*Aspergillus terreus*	*Carthamus lanatus*	Antimalarial, antimicrobial, and anti-leishmanial activities.	[98]
Flavonoids—derived Metabolites				
Apigenin	*Colletotrichum* sp.	*Ginkgo biloba*	Antidiabetic, antioxidant, anticancer, and Antibacterial activities	[133,134,135,136]
	*Chaetomium globosum*	*Cajanus cajan*		
	*Paraconiothyrium variabile*	*Cephalotaxus harringtonia*		
Cajanol	*Hypocrea lixii*	*Cajanus cajan*	Antimicrobial and Anticancer activities.	[137]
Chrysin	*Alternaria alternata*	*Passiflora incarnata*	Anticancer activity	[138]
	*Colletotrichum capsici*			
	*Colletotrichum taiwanense*			
Chalcone	*Ceriporia lacerata*	*Cleistocalyx operculatus*	Anti-inflammatory, Antibacterial, antitumor, and antifungal activities.	[139]
Curcumin	*Chaetomium globosum*	*Curcuma wenyujin*	Antioxidant, antitumor, and Anti-inflammatory activities.	[140]
Quercetin	*Aspergillus nidulans*	*Ginkgo biloba*		[141,142,143]
	*Aspergillus oryzae*			
	*Annulohypoxylon squamulosum*	*Cinnamomum sp.*		
	*Nigrospora oryzae*	*Loranthus micranthus*		
Kaempferol	*Fusarium chlamydosporum*	*Tylophora indica*	Antitumor, Antibacterial, anti-inflammatory, antioxidant, and antidiabetic activities.	[142,144,145,146]
	*Mucor fragilis*	*Podophyllum hexandrum*		
	*Annulohypoxylon boveri var. microspora*	*Cinnamomum sp.*		
	*Annulohypoxylon squamulosum*			
Rotenone	*Penicillium* sp.	*Derris elliptica*	Pesticide and Insecticide activities.	[147]
Silymarin	*Aspergillus iizukae*	*Silybum marianum*	Hepatoprotective, cardioprotective, anti-inflammatory, antioxidant, and Anticancer activities.	[148]
Vitexin	*Dichotomopilus funicola*	*Cajanus cajan*	Neuroprotective, antitumor, and Antioxidant activities.	[135,149]
	*Colletotrichum* sp.	*Ginkgo biloba*		
Rutin	*Chaetomium* sp.	*Nerium oleander*	Neuroprotective, cardioprotective, and Antioxidant activities.	[150,151,152]
	*Xylaria* sp.	*Ginkgo biloba*		
	*Aspergillus flavus*	*Aegle marmelos*		
Luteolin	*Aspergillus fumigatus*	*Cajanus cajan*	Antioxidant activity	[153]
Hybrid and Other types of metabolites				
Torreyanic acid	*Diaporthe* sp.	*mangroves*	Cytotoxic activity	[154]
Diaportheone B	*Pestalotiopsis microspora*	*Torreya taxifolia*		[50]
Chaetoglobosin A	*Alternaria* sp.	*Polygonum senegalense*		[155]
Cytochalasin E	*Chaetomium globosum*	*Panax notoginseng*		[156]
Indole-3-carboxylic acid	*Chaetomium* sp.	*Otanthus maritimus*		[83]
Cytochalasin Z28	*Xylaria hypoxylon*	*Piper aduncum*		[157]
	*Penicillium chrysogenum*	*Cistanche deserticola*	Neuroprotective activity	[51]
Chaetoglobosin A *	*Chaetomium globosum* CDW7	*Ginkgo biloba*	Antifungal activity	[158]
Chaetoglobosin D				
2-amino-3,4-dihydroxy-2-25-(hydroxymethyl)-14-oxo-6,12- eicosenoic acid *	*Mycosphaerella* sp.	*Eugenia bimarginata*		[159]
Myriocin				
Microsphaerol	*Microsphaeropsis* sp.	*Salsola oppositifolia*		[160]
2(2-hydroxyphenyl) acetic acid *				
2-phenylethyl 1H-indol-3-yl-acetate				
Phomopsichalasin	*Diaporthe* sp. P133	*Pandanus amaryllifolius*	Antibacterial Activity	[161]
Fusaruside	*Fusarium* sp. IFB-121	*Quercus variabilis*		[162]
2S,20R,3R,30E,4E,8E)-1-O-beta-Dglucopyranosyl-2-N-(20 -hydroxy-30-octadecenoyl)-3-hydroxy-9-methyl4,8-sphingadienine *				
Cytochalasin D	*Aspergillus clavatus*	*Taxus mairei*	Antiangiogenic activity	[4]
Cytochalasins E	Phomopsis sp.		Anti-microbial activity	
Cytochalasins K	Plectophomella sp.	-	Antifungal, Antibacterial, and Herbicidal Activities	[99]
	Physalospora sp.			
(6S,2E)-6-hydroxy-3-methoxy-5-oxodec-2-enoic acid	*Pestalotiopsis* sp. DO14	*Dendrobium officinale*	Cytotoxic and Antifungal Activities	[126]
3-(2,3-dihydroxyphenoxy)-butanoic acid	*Nodulisporium* sp.	*Erica arborea*	Antialgal and Antifungal activities	[96]
5-hydroxymethylfurfural	*Botryosphaeria dothidea* KJ-1	*Melia azedarach*	Cytotoxic, Antibacterial, Antifungal, and Antioxidant activities	[88]

* Denote structure is not available in the 1–7 structure figures.

**Table 2:** Compiled list of existing AI-based tools enabling NPs discovery.

AI Tool	AI Technology	Advantages	Limitations	Application	References
Genome Mining and Biosynthetic Gene Cluster (BGC) Prediction					
antiSMASH	BGC detection using ML-supported heuristics.	- The most widely used platform for BGC detection	- Limited discovery of novel BGCs	- Used to identify the BCGs of Beauvericin and Oxepinamide - Identify hybrid cytochalasin PKS–NRPS BCG in fungal genome	[191,192,193]
PRISM	Predicts and identifies NP scaffolds from BGC using ML models	- Works well with fungal PKS/NRPS pathways.	- Limited discovery of novel BGCs	- Predict NRPS-derived peptide scaffolds related to etamycin.	[194,195]
ClusterFinder	Use Hidden Markov Models (HMM)-based generative probabilistic modeling to detect noncanonical fungal and bacterial BGCs.	- Detect novel classes of BGCs.	- High rate of false positives	- Human microbiome mining for BCGs is associated with antibiotics.	[185,186]
DeepBGC	Use the Deep Learning (using Pfam2vec and Random Forest classifiers) algorithm.	- Detect non-canonical and novel BGCs.	- High rate of false positives	- Prediction of a wide range of BGCs, including NRPS, PKS, and terpene	[187,196]
decRiPPter	Support Vector Machines (SVM)/Machine Learning algorithms.	- Predict novel families of (RiPPs).	- Higher rate of false positives.	- Discovery of Pristinin A3	[197]
DeepRiPP	Deep Learning (Natural language processing).	- Enables the non-canonical RiPPs. - Higher discovery rate	- Limited to RiPPs	- Discovery of Deepflavo, deepginsen, and deepstreptin.	[198]
GECCO	Deep Learning (CRF-based)	- Detect novel classes of BGCs. - Predict potential bioactivity.	- High false positive rate.	- Metagenome mining for novel clusters	[199]
DRNMs	These models use deep learning to predict transcription factor binding and regulatory elements controlling BGC expression.	- Detect complex, nonlinear interactions. - Multi-omics data integration.	- High false positive rate.	- Predication silent BCGs and metabolite production. - Integrate a wide range of omics data.	[200]
AI-Assisted Metabolomics and MS/MS Analysis and Structure Elucidation					
GNPS	It uses machine learning on MS/MS spectral networks to cluster structurally related metabolites. Employ feature-based molecular networking (FBMN)	- Dereplication using community spectral libraries. - Identify analog families through molecular networking.	- High false positive rate.	- Molecular networking for NPs. - Dereplication of known NPs.	[201,202]
SMART 2.0	Convolutional Neural Networks (CNN) for structure elucidation	- Guides discovery and structure elucidation	- Limited coverage of all possible protein families.	- Discovery of novel chimeric macrolide, Symplocolide A	[203]
DP4-AI	Bayesian Approach combined with Quantum Chemistry	- Automates NMR shift calculations	- Exploration of conformational space is computationally demanding for flexible molecules.	- Stereochemistry determination—Automated structure elucidation.	[204]
ASE-ANI	Machine Learning	- Filters force field-generated conformations - Reduce the high computational cost of quantum chemistry calculations.	- High false positive rate.	- Generate a wide range of conformers and rank them for docking and NMR shift prediction.	[205]
COLMAR	AI/Database Matching	Identifies and annotates primary metabolites from the NMR spectra of complex mixtures.	- Require 2D NMR spectra - Overlapped spectra in complex mixtures - Detection of known metabolites only.	- Identify primary metabolites from a complex NMR spectrum	[206]
SIRIUS + CSI	Fragmentation-tree algorithms Deep neural networks to predict structural fingerprints and candidate structures	- High accuracy for complex fungal metabolites. - Combined with GNPs for molecular networking integration.	- Limited capacity distinguishing highly similar isomers or stereoisomers.	- Determination of molecular formula - Screening of complex fungal metabolites	[207]
MS2LDA	Applies topic modeling (LDA) to MS/MS substructure motifs	- Independent database discovery. - Handle large sets of MS/MS sets.	- Limited full structure elucidation - No bioactivity prediction	- Identify azaphilone-related substructures and to group known and putative azaphilone analogs within complex MS/MS datasets	[208]
AI-assisted for Target identification and Bioactivity Prediction					
Chemprop	A graph neural network model for predicting physicochemical and biological properties of NPs.	- Predicts ADMET, bioactivity, and drug-likeness of microbial metabolites. - Supports prioritization of NP leads.	- Limited full structure elucidation. - Require highly diverse training data.	- Prediction of antifungal activity of polyketides. - QSAR modeling of fungal alkaloids. - Toxicity prediction of NP analogs.	[209]
SPIDER	Utilize Self-Organizing Maps (SOM) algorithms	- Predict bioactivities. - Predict mutation and detect variants in peptide structures.	- Limited to proteomic analysis. - Limited capacity for discovery of novel proteins in homologous sequence sets	- Predict the biological activity of macrocyclic natural NPs and fragment-like NPS	[210]
Generative AI and In Silico NP Design					
Generative Models (Original Tool)	Generative models (GANs, VAEs, diffusion models) create NP-like scaffolds.	- Exploring novel scaffold - Generating rapid hypotheses before synthesis	- Limited capacity for discovery of novel molecules - A generated scaffold does not guarantee biosynthetic feasibility.	- Generative models used to propose coumarin derivatives, polyketide-like scaffolds, and naphthoquinone analogs,	[211,212]
Molecular Diffusion Models for NP Design	Diffusion-based models generate novel NP scaffolds with improved realism and chemical diversity.	- Explore novel analogs of NPS and propose structure modification. - Working with 3D coordinates, which is useful in the case of complex NB	- Large and diverse chemical datasets needed - Limited biosynthetic feasibility.	- Limited capacity in predicting meroterpenoid analog and PKS-like polyketide design.	[213,214]
AI-assisted tool for Protein Structure Prediction					
AlphaFill for Substrate-Bound Models	enhances AlphaFold models by adding cofactors, substrates, or ligands.	- Ligand addition to the predicted proteins. - Provide information about active sites and docking - Processing multiple protein structures at once.	- Low predication for substrate specificity.	- Design enzyme-substrate interactions. - Metabolic enzymes structural annotation	[215]
AI-assisted tool Microbial Networking and Ecology					
Microbiome ML Co-Occurrence Models	These models detect ecological patterns linked to NP biosynthesis.	- Ability to capture interactions at the community level. - Complex dataset handling and guide NP discovery.	- Low resolution for revealing all levels of microorganisms. - Environmental effects.	- Design ecological mapping for microbial communities. - Predict endophyte-host interaction.	[216]
AI-assisted tool Synthetic Biology & BGC Engineering					
RetroBioCat AI	Machine-learning tools for retrosynthesis and biosynthesis route design and enzyme selection.	- Generates optimal biosynthetic pathways for NP analog synthesis. - Predicts enzyme candidates for pathway refactoring.	- Limited capacity to predict the full kinetic and efficiency of the reaction. - Prediction depends on the rule of known enzymes	- Design of fungal phenylpropanoid derivatives. - Enzyme redesign for meroterpenoid biosynthesis. - Optimization of NRPS/PKS pathway steps.	[217]

AI: Artificial intelligence/ML: Machine learning/NP: Natural product/HMM: Hidden Markov Models/decRiPPter: Data-driven Exploratory Class-independent RiPP Tracker/RiPPs: ribosomally synthesized and post-translationally modified peptides/DRNMs: Deep Regulatory Network Models/PRISM: Prediction Informatics for Secondary Metabolomes/QSAR: Quantitative Structure-Activity Relationship/NRPS: Nonribosomal Peptide Synthetases/PKS: Polyketide Synthases.

## Data Availability

No new data were created or analyzed in this study. Data sharing is not applicable to this article.
